# Diagnostic accuracy of reused Pronto Dry® test and CLOtest® in the detection of *Helicobacter pylori* infection

**DOI:** 10.1186/s12876-015-0332-0

**Published:** 2015-08-12

**Authors:** Shahidi Jamaludin, Nazri Mustaffa, Nor Aizal Che Hamzah, Syed Hassan Syed Abdul Aziz, Yeong Yeh Lee

**Affiliations:** 1 School of Medical Sciences, Universiti Sains Malaysia, Kubang Kerian, Kelantan Malaysia; 2 Pasir Gudang Specialist Hospital, Pasir Gudang, Johor Malaysia

## Abstract

**Background:**

Unchanged substrate in a negative rapid urease test may be reused to detect *Helicobacter pylori* (*H. pylori*). This could potentially reduce costs and wastage in low prevalence and resource-poor settings. We thus aimed to investigate the diagnostic accuracy of reused Pronto Dry® and CLOtest® kits, comparing this to the use of new Pronto Dry® test kits and histopathological evaluation of gastric mucosal biopsies.

**Methods:**

Using a cross-sectional study design, subjects who presented for upper endoscopy due to various non-emergent causes had gastric biopsies obtained at three adjacent sites. Biopsy samples were tested for *H. pylori* using a reused Pronto Dry® test, a reused CLOtest®, a new Pronto Dry® test and histopathological examination. Concordance rates, sensitivity, specificity, positive predictive value (PPV), negative predictive value (NPV) and diagnostic accuracy were then determined.

**Results:**

A total of 410 subjects were recruited. The sensitivity and diagnostic accuracy of reused Pronto Dry® tests were 72.60 % (95 % CI, 61.44 – 81.51) and 94.15 % (95 % CI, 91.44 – 96.04) respectively. For reused CLOtests®, the sensitivity and diagnostic accuracy were 93.15 % (95 % CI 85.95 – 97.04) and 98.29 % (95 % CI 96.52 – 99.17) respectively. There were more true positives for new and reused Pronto Dry® pallets as compared to new and reused CLOtests® when comparing colour change within 30 min vs. 31–60 min (*P* < 0.001 and *P* = 0.7 respectively).

**Conclusion:**

Negative Pronto Dry® and CLOtest® kits may be reused in a low prevalence setting where cost issues remain paramount. Reused CLOtest® kits have better accuracy than reused Pronto Dry® tests. Reused Pronto Dry® tests however have a more rapid colour change whilst maintaining diagnostic accuracy.

## Background

Since its identification in 1982 by Barry Marshall and Robyn Warren, the flagellated Gram-negative bacilli *Helicobacter pylori* (*H. pylori*) has been recognised as the predisposing factor for gastroduodenal diseases, particularly peptic ulcer disease, gastric malignancies and B-cell mucosa-associated lymphoid tissue (MALT) lymphomas [1–3]. Due to the availability of commercial test kits as well as solutions prepared in-house, the rapid urease test (RUT) has been widely used for the detection of *H. pylori*, with reported studies indicating high sensitivity (95–98 %) and specificity (92–100 %) [4–7]. RUT assays exploit the fact that *H. pylori* produces large amounts of urease, which hydrolyses urea to ammonia thus enabling the organism to survive in a low pH environment [8, 9].

Theoretically, unchanged substrate in a negative RUT may be used again to test for the presence of *H. pylori* in a new gastric biopsy sample. This has been shown to be true for CLOtest® pallets, where some studies have concluded that diagnostic accuracy is maintained despite being reused up to 6 months from the initial test application [10–14]. The CLOtest® has a urea gel capsule that changes colour over time in the presence of specimens that contain *H. pylori*, whilst the Pronto Dry® kit consists of a dry filter paper that contains urea and an indicator that detects a rise in pH of specimens that are *H. pylori* positive. The main advantages of using Pronto Dry® over CLOtest® kits in the detection of *H. pylori* is the fact that Pronto Dry® kits may be stored at room temperature, with no need to incubate the applied specimen in a warm environment thus offering quicker results. There is also a linear correlation between the histological grading of *H. pylori* stomach mucosal colonisation density with the Pronto Dry® graded colour change index. In comparison to the various studies done on the reuse of CLOtest® kits, there is a paucity of information in the literature on the ability of reused Pronto Dry® tests to detect the presence of *H. pylori*. Bearing in mind the issue of potential cost-savings and reduction in wastage especially in a resource-poor setting, we thus sought to assess the diagnostic accuracy of re-using negative Pronto Dry® test and CLOtest® kits in comparison to *H. pylori* detection using new Pronto Dry® test kits as well as histological methods.

## Methods

### Study participants

This cross-sectional study was performed from March 2008 to June 2010 in Hospital Universiti Sains Malaysia, Kubang Kerian. Kelantan. This is a tertiary referral centre and a teaching hospital located at the northeastern region of Peninsular Malaysia. Inclusion criteria were subjects aged 18 and above who presented for upper GI endoscopy due to investigation of upper abdominal symptoms or suspected gastric pathology. Exclusion criteria were subjects who had recently taken antibiotics, bismuth salts and/or proton pump inhibitors within 2 weeks of their endoscopy date, were pregnant, had overt upper GI bleeding as well as those who could not tolerate upper gastrointestinal endoscopic examination.

Eligible participants were provided with an information sheet that contained relevant details of the study. A verbal explanation was also provided and informed consent obtained. Following this, clinical and demographic details of participants were recorded. Participants would then undergo upper GI endoscopy as per standard protocol, where after adequate endoscopic examination gastric biopsies would then be obtained for assessment of *H. pylori* infection. Four separate biopsies, each measuring approximately 2 – 3 mm were obtained using the same biopsy forceps from the same adjacent gastric area for *H. pylori* testing using a new Pronto Dry® kit, a reused Pronto Dry® kit, a reused CLOtest® as well as for histopathology assessment. Histological examination was performed by pathologists who were blinded to study outcomes where *H. pylori* was reported to be either present or absent. Relevant histological details were reported using the Sydney classification.

### Ethical approval

Approval was obtained from the Human Research Ethics Committee (HREC), Universiti Sains Malaysia (Ref.: USMKK/PPP/JEPeM [200.4(2.5)]).

### New and reused Pronto Dry® kits

The new as well as reused Pronto Dry® kits for this study were produced by Medical Instruments Corporation, France; and reused pallets that were used in this study varied from an interval period of 1–28 weeks after the first negative interpretation. Both new and reused Pronto Dry® kits were stored at room temperature (20–25 °C). There was no special precaution taken prior to using a new Pronto Dry® kit, however for a reused Pronto Dry® kit (i.e. no colour change at 1 h after the first use), the date of first use was noted prior to applying the second specimen adjacent to the previous tissue biopsy sample. The test kits were read at 1, 10, 15, and 30 min, then 1, 12 and 24 h, with the time taken for a positive result for each test kit recorded.

### Reused CLOtest® kits

The reused CLOtest® in this study was manufactured by Ballard Medical Products, Utah, USA. A reused CLOtest® was one that had been used once previously but with a negative result after 24 h. The time interval between initial use and reuse of the pallets ranged from 1 to 24 weeks. These kits were stored in the refrigerator at 5 °C whilst awaiting reuse. The label for a reused kit was lifted far enough to expose the yellow gel to room temperature before second usage. For quicker test results, we allowed the gel to reach room temperature between 7 and 10 min prior to inserting the new biopsy specimen.

### Data and statistical analysis

Sample size was calculated based on sensitivity and specificity of reused CLOtest® kits in a study by Lee et al. [10]. For a study power of 80 % and α of 0.05, 369 subjects were needed. Another 10 % was added to account for possible attrition, giving a final number of 410 subjects. The Statistical Package for Social Sciences Ver. 18 (SPSS Inc., Chicago, Illinois, USA), as well as OpenEpi Ver. 2.3 [15] was used for data entry and statistical analysis. Categorical variables were expressed as frequency with percentage and continuous data as mean with standard deviation (SD). For comparison purposes, *H. pylori* was considered present if either the new Pronto Dry® test or histology was positive and thus regarded as the ‘standard test’ in the current study. Performance of reused rapid urease tests vs. standard tests was assessed for sensitivity, specificity, positive predictive value (PPV), negative predictive value (NPV), accuracy and likelihood ratio (LR) at 95 % confidence intervals (CIs). Concordance between reused vs. new tests was assessed using the Kappa agreement test. A *P* value ≤ 0.05 was considered clinically significant.

## Results

A total of 410 participants who presented between March 2008 and June 2010 for upper GI endoscopic assessment were recruited into this study. There were 236 male and 174 female participants (57.6 and 42.4 % respectively), with a mean age of 54.1 ± 15.4 years. The majority of participants who underwent upper endoscopy was for investigation of recurrent dyspepsia (*n* = 205, 41.2 %), whilst 133 (32.4 %) underwent an endoscopic reassessment due to a previous episode of upper GI tract bleed. Upon histological examination, 244 (59.5 %) participants were reported to have chronic gastritis, 124 (30.2 %) with gastric ulcers, 121 (29.5 %) with duodenal ulcers and 119 (29 %) had erosive oesophagitis. Other findings included gastroduodenitis, uraemic gastropathy and oesophageal varices with one case of gastric carcinoma. The overall prevalence of *H. pylori* infection was low i.e. 17.8 % (73/410) based on either a positive new Pronto Dry® test kit (68/73) or histology (73/73).

### Diagnostic accuracy of reused Pronto Dry® test

**Fig. 1:**
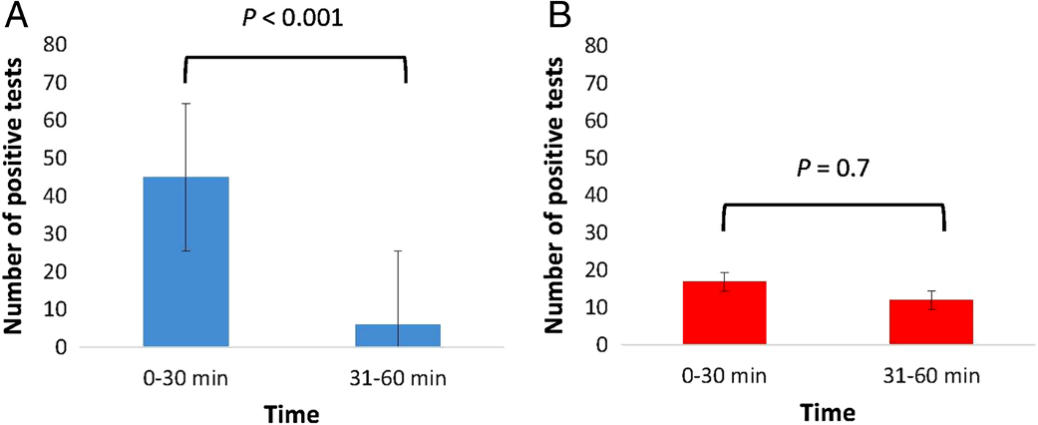
Colour change time for 0–30 min vs. 31–60 min for reused Pronto Dry® (**a**) and reused CLOtest® (**b**) when paired with the new Pronto Dry® pallets (i.e. true positives), # *P* value significant if < 0.05

For reused Pronto Dry® test vs. standard test, 53 tested positive and 333 were negative. Thus the concordance rate of reused Pronto Dry® tests was 94 % (*n* = 386) with the remaining 24 (5.8 %) having discrepant results. The discrepant results were observed in 20 reused Pronto Dry® negative pallets (i.e. false negatives) and four in reused Pronto Dry® positive pallets (i.e. false positives). Therefore, the sensitivity and specificity of the reused Pronto Dry® test kits were 72.60 % (95 % CI 61.44 – 81.51) and 98.81 % (95 % CI 96.99 – 99.54). The PPV was 92.98 % (95 % CI 83.30 – 97.24) and NPV was 94.33 % (95 % CI 91.41 – 96.30) with a diagnostic accuracy of 94.15 % (95 % CI 91.44 – 96.04) (Table 1). While the LR of a positive reused test is good i.e. 61.17 (95 % CI 36.95 – 101.20), the LR of a negative test is low i.e. 0.27 (95 % CI 0.25 – 0.31). The kappa agreement between reused Pronto Dry® and standard test at 1 h was 0.78 (95 % CI, 0.69 – 0.88). The majority of the reused Pronto Dry® pallets that were positive for *H. pylori* (88.7 % or *n* = 47/53) took less than 30 min to change colour, with 11.3 % (*n* = 6/53) taking more than 30 min. In less than 30 min, 45 of new and reused Pronto Dry® pallets turned positive compared to 2 of new and reused pallets in 31 – 60 min; this was significantly different (*P* < 0.001) (Fig. 1).

**Table 1:** Sensitivity, specificity, predictive values, diagnostic accuracy and likelihood ratios of reused Pronto Dry® test and reused CLOtest® for *H. pylori* infection

Parameter	Reused Pronto Dry® % (95 % CI^a^)		Reused CLOtest® % (95 % CI^a^)	
Sensitivity	72.60 %	(61.44, 81.51)	93.15 %	(84.95, 97.04)
Specificity	98.81 %	(96.99, 99.54)	99.41 %	(97.86, 99.84)
Positive predictive value	92.98 %	(83.30, 97.24)	97.14 %	(90.17, 99.21)
Negative predictive value	94.33 %	(91.41, 96.30)	98.53 %	(96.60, 99.37)
Diagnostic accuracy	94.15 %	(91.44, 96.04)	98.29 %	(96.52, 99.17)
Likelihood ratio of a positive test	61.17	(36.95 – 101.20)	157	(58.78 – 419.1)
Likelihood ratio of a negative test	0.27	(0.25 – 0.31)	0.07	(0.05 – 0.10)
Diagnostic odds	220.60	(72.56 – 670.70)	2278	(432.9 – 11990)
Cohen’s kappa (Unweighted)	0.78	(0.69 – 0.88)	0.94	(0.84 – 1.03)

^a^Wilson score interval is used for estimation of 95 % confidence interval (95 % CI)

### Diagnostic accuracy of reused CLOtest®

For reused CLOtest® kits, 68 were reported as positive and 335 negative. The concordance rate was 98 % (*n* = 404) with 6 discrepant results, where 4 were observed in reused CLOtest® negative pallets (i.e. false negatives) and two in reused positive pallets (i.e. false positives). The sensitivity and specificity of reused CLOtest® kits were 93.15 % (95 % CI 84.95 – 97.04) and 99.41 % (95 % CI, 97.86 – 99.84) (Table 1). The PPV was 97.14 % (95 % CI 90.17 – 99.21) and NPV was 98.53 % (95 % CI 96.60 – 99.37) with a diagnostic accuracy of 98.29 % (95 % CI 96.52 – 99.17). The kappa agreement between reused CLOtest® vs. standard test at 24 h was 0.94 (95 % CI, 0.84 – 1.03). Most of the reused CLOtest® pallets took 31–60 min to change colour, indicating presence of *H. pylori* (44.1 % or 33/68 of the positive samples). For reused CLOtest® and new Pronto Dry® pallets, 17 became positive within 30 min whilst 12 took between 30 and 60 min. This however was not significantly different (*P* = 0.7) (Fig. 1).

## Discussion

Various methods of *H. pylori* detection, either invasive or non-invasive in nature are available with each of them having different diagnostic accuracies. There have been many studies comparing these detection methods to ensure superiority in terms of diagnostic accuracy and qualitative characteristics between them. Cost however is always an issue to be reflected upon, especially in resource-poor circumstances. As it costs approximately $6 for a new Pronto Dry® or CLOtest® kit, reusing negative kits may bring about substantial cost savings. In fact, VH Chong calculated that at a CLOtest® price of $5.72 per kit a potential cost saving of $2.45 per patient was achievable with maximal kit reuse. This translated to an annual cost savings of $2,941/year based on their patient population [13]. Also, based on the fact that unaltered substrate that has not been consumed in a negative rapid urease test may be reused, studies were done to assess the feasibility of re-using CLOtest® pallets. Results were encouraging, proving that these reused pallets had a high diagnostic accuracy with the ability to be used repeatedly over a period of months following an initial negative CLOtest® result. To our knowledge however, there is no published data on the diagnostic accuracy and qualitative characteristics of reused Pronto Dry® kits.

In our cross-sectional study of 410 adults who underwent upper GI endoscopy in a non-emergent setting, we found that the overall prevalence of *H. pylori* infection was low at 17.8 % (73/410). This prevalence was based on the results of using either a new Pronto Dry® kit or a positive histology examination for *H. pylori*, and is slightly higher compared to results quoted by Gurjeet and Naing (prevalence of 13.5 %) [16] but nevertheless reflecting the general prevalence of *H. pylori* infection in the northeastern coast of Peninsular Malaysia [17]. In comparison, based on rapid urease tests a much higher prevalence of *H. pylori* infection (49.0 %) was recorded among dyspeptic Malaysians in highly developed Kuala Lumpur, the capital city of Malaysia [18]. This disparity in *H. pylori* prevalence has been noted before in multiple studies, but despite theories ascribing this to differences in ethnicity and genetics as well as related socioeconomic plus dietary factors, no definite answer has been found [19–22]. An exceptionally low prevalence of *H .pylori* as seen in this population may mean possible wastage (and associated high costs) if an RUT is used for each and every patient during endoscopy. Reusing these tests could therefore be a preferable choice in a low prevalence and low resource setting.

With regards to re-using rapid urease tests, earlier studies have shown that reused CLOtest® kits have similar sensitivity and specificity as compared to new ones [10, 12]. Our results support the validity of reusing CLO tests. On the other hand, reused Pronto Dry® had demonstrated a relatively lower sensitivity (72.60 %) in the detection of *H. pylori*, but specificity remained high at 98.81 %, with a diagnostic accuracy of 94.15 %. Several factors or limitations were identified that could have explained this difference in sensitivity for reused Pronto Dry® test. For instance, the time interval from initial use to re-use of the Pronto Dry® kit may have been too far apart, ranging from 7 days up to 7 months. Reagents may have deteriorated over this period, resulting in the high number of false negative results seen. At the same time due to the presence of the previous specimen there may be less media left for implantation on to the urea soaked paper of a reused Pronto Dry® test kit, leading to a poor chemical reaction. Alternatively there may be issues in sampling as *H. pylori* does not uniformly colonise the stomach lining causing varying population densities. Recent PPI use may also give a negative result; we however have excluded such patients from our study. Likelihood ratios (LR) may be used to compare between two diagnostic methods, and in our study (as stated in Table 1) there were good LR for positive reused Pronto Dry® and reused CLOtest® kits (61.17 and 157 respectively). As for the reused kits with negative results, LR for reused Pronto Dry® was 0.27 whilst for a reused CLOtest® this was 0.07 indicating that a reused CLOtest® was much better at indicating the absence of *H. pylori*.

Many factors affect the time taken for colour change in RUTs which include the urease testing process itself (warmed vs room temperature), bacterial load, biopsy location (antrum vs body), biopsy size as well as number of specimens [8, 23]. In our study 88 % of the positive reused Pronto Dry® pallets changed colour within 30 min, which was similar in duration to new Pronto Dry® tests. However, this was not the case with reused CLOtest®. Previous studies on the qualitative characteristic of new Pronto Dry® tests have been done [24], which showed that a new Pronto Dry® test offers a quicker diagnosis as compared to a new CLOtest® kit at 30 min with its chromatin grading correlating well with stomach mucosal *H. pylori* colonisation density. Hence we can conclude that the chemical in reused Pronto Dry® kits are still able to maintain their qualitative characteristics similar to a new test kit with regards to colour changing time of up to a seven month period. The same cannot be said for reused CLOtest® kits. Another main difference between reusing CLOtest® vs Pronto Dry® kits is the fact that the former needs to be in refrigerated storage prior to use, and then brought to room temperature prior to inserting the biopsy specimen. Pronto Dry® kits on the other hand have a practical advantage as it can be stored at room temperature and used immediately when needed. It must be cautioned though that a tropical climate might contribute to a more rapid deterioration of used Pronto Dry® and CLOtest® kits, as well as having a higher possibility of fungal growth and contamination.

## Conclusion

Our study showed that reused Pronto Dry® test kits have a relatively lower diagnostic accuracy as compared to new Pronto Dry® test kits. Reused CLOtest® kits however were able to maintain its diagnostic accuracy. Plausible explanations or limitations for this study include a prolonged interval between initial use and reuse and a decreased media for specimen implantation. On the other hand, reused Pronto Dry® rather than CLOtest® offer a quicker diagnostic result despite being stored for a prolonged period. Therefore if accuracy is of concern a reused CLOtest® is preferable, alternatively a reused Pronto Dry® test may be a better choice for quick and reliable results.
